# Integrating Advance Care Planning into End-of-Life Education: Nursing Students’ Reflections on Advance Health Care Directive and Five Wishes Assignments

**DOI:** 10.3390/nursrep15080270

**Published:** 2025-07-28

**Authors:** Therese Doan, Sumiyo Brennan

**Affiliations:** 1 School of Nursing, San Francisco State University, San Francisco, CA 94132, USA; 2 Institute for Gerontology, J.F. Oberlin University, Tokyo 3758, Japan; sumiyo@sfsu.edu

**Keywords:** advance care planning, end-of-life education, advance health care directive, five wishes, experiential learning, reflective assignment, nursing

## Abstract

**Background/Objectives**: End-of-life care is a vital part of nursing education that has been overlooked until recent years. Advance care planning should be incorporated into the prelicensure nursing curriculum to build student nurses’ confidence in aiding patients and families with their preferred future care plans. Advance care planning tools, such as the Advance Health Care Directive (AHCD) and Five Wishes, provide experiential learning opportunities that bridge theoretical knowledge with real-world patient advocacy. In this study, students were asked to complete either the AHCD or Five Wishes document as though planning for their own end-of-life care, encouraging personal reflection and professional insight. Embedding these assignments into nursing education strengthens students’ confidence in facilitating end-of-life discussions. This study applied Kolb’s experiential learning theory, including concrete experience, reflective observation, abstract conceptualization, and active experimentation, to explore student nurses’ perspectives on the Advance Health Care Directive and Five Wishes assignments, as well as their understanding of end-of-life care. **Methods**: This study used an exploratory–descriptive qualitative design featuring one open-ended question to collect students’ views on the assignments. **Results**: The final sample comprised 67 prelicensure student nurses from Bachelor of Science and Entry-Level Master’s programs. The Advance Health Care Directive and/or Five Wishes assignment enhanced students’ understanding of end-of-life decision-making. **Conclusions**: It is essential to complete the assignment and immerse oneself in an end-of-life situation to grasp patients’ perspectives and concerns regarding when to engage in difficult conversations with their patients.

## 1. Introduction

In the *Essentials*, the American Association of Colleges of Nursing recommends that the framework for preparing the future nursing workforce includes ten competencies that should be applied throughout the human lifespan, across four spheres of care, and for diverse patient populations [1]. This study focuses on a nursing course titled Chronic Care and End-of-Life Care (EOLC), which falls under one of the four spheres, specifically hospice and palliative care.

Understanding EOLC is a vital component of nursing education [2]. Nurses play a central role in providing compassionate, patient-centered care during the final stages of life and support patients and families through complex decision-making [3]. EOLC is comprehensive and may encompass primary care, spiritual care, care coordination, and the effective management of pain and symptoms. It involves a multidisciplinary approach that includes support for the patient’s family and significantly impacts the patient’s quality of life (QOL) [2,4]. EOLC could be taught through various modalities, including didactic instruction, clinical experiences, simulation, and activity-based assignments [5,6]. These educational strategies should emphasize communication skills, ethical considerations, and symptom management.

Addressing knowledge gaps, a lack of confidence, and limited practical skills through education, mentorship, interprofessional collaboration, and evidence-based practice enables nurses to deliver compassionate and patient-centered EOLC [7]. While in nursing school, it is essential for students to become familiar with medical and legal documents related to EOLC, such as the Advance Health Care Directive (AHCD), Five Wishes, and Physician Orders for Life-Sustaining Treatment (POLST) [8,9].

Specifying preferences for EOLC is both important and meaningful for patients and families. Although nurses in the U.S.A. are generally more comfortable with EOLC than their peers in other countries, critical issues remain, particularly a lack of education and confidence in facilitating these conversations. The root of this issue lies in insufficient training in the extant nursing curricula, with students reporting a lack of confidence when faced with EOLC scenarios [7,10,11].

### 1.1. Importance of EOLC Preparation for Prelicensure RN Students

There is widespread agreement on the importance of improving EOLC preparation for student nurses [12]. Despite their commitment, many student nurses feel unprepared to address palliative care and/or EOLC during their education. This lack of preparation often leads to emotional distress and a sense of helplessness when faced with death and dying [13]. While emotionally challenging for all students, including key concepts of EOLC in nursing education can positively shape their attitudes and increase their comfort with these discussions [14]. Their understanding and perspective are crucial in ensuring that patients and families receive appropriate and quality care and support during critical moments [15,16]. Preparing prelicensure students for EOLC is essential not only for improving patient outcomes but also for strengthening students’ professional development and readiness for clinical practice [14].

### 1.2. Advance Care Planning

Advance care planning (ACP) involves engaging in discussions and making informed decisions about future medical care, particularly in the event of a severe illness or inability to communicate one’s preferences. At the heart of ACP is a commitment to meaningful conversations between individuals, loved ones, and health care professionals to ensure patients’ values and preferences are respected [17,18].

One common form of ACP documentation is the Advance Health Care Directive (AHCD), a legal document that outlines a person’s EOLC preferences, including medical interventions to be followed if they cannot speak for themself [19]. The most common types of AHCDs are the Living Will and the Durable Power of Attorney for Healthcare [20]. Additional ACP tools include Physician Orders for Life-Sustaining Treatment (POLST), and other equivalents such as Orders for Scope of Treatment (POST), Medical Orders for Scope of Treatment (MOST), and Transportable Physician Orders for Patient Preference (TPOPP) [19,21].

As an alternative to the AHCD, individuals may complete the Five Wishes (5W) document, a user-friendly and holistic tool that combines the legal and emotional aspects of EOLC planning. Created in 1996 by a non-profit organization in Florida, the 5W document integrates health care surrogacy with a living will and reflects the patient’s physical, emotional, and spiritual needs [22]. A national version was released in 1998 with the support of the American Bar Association’s Commission on Law and Aging. Developed with input from health care professionals, legal experts, and spiritual leaders, the 5W is accessible to people of all ages and cultural backgrounds [23]. Since then, it has become one of the most widely used AHCD documents in the U.S., supported by trusted organizations like AARP and the American Bar Association. The 5W is now available in over 30 languages and is used by millions globally to ensure their EOLC wishes are honored [22,24]. While 5W is widely used and recognized across all 50 states, its legal validity varies, and in some states, it may need to be accompanied by additional forms to meet statutory requirements [22].

This study examined the perspectives of prelicensure nursing students on end-of-life care (EOLC) through the lens of the Advance Health Care Directive (AHCD) and Five Wishes (5W) assignments completed during their final semester of nursing education.

## 2. Materials and Methods

### 2.1. Conceptual Model

This study is guided by Kolb’s Learning Cycle, a four-stage model of learning that includes concrete experience, reflective observation, abstract conceptualization, and active experimentation [25,26]. Kolb’s framework provides a structured pedagogical foundation for understanding how nursing students process complex health care concepts such as EOLC. In this context, the experiential assignment asked students to complete either an AHCD or a 5W document as though they were planning for their own end-of-life care. The aim was to use reflective learning to introduce students to the emotional and practical realities of advance care planning (ACP). By placing themselves in the role of a patient making EOLC decisions, students had the opportunity to gain both personal insight and professional awareness about medical decision-making when capacity is lost. Research suggests that nurses who complete their own AHCDs are more likely to recommend ACP to others and initiate such conversations with patients [27]. Completing either the AHCD or 5W serves as a form of self-examination and self-awareness, key competencies in compassionate, patient-centered care. Table 1 presents the AHCD/5W assignment through the lens of Kolb’s Learning Cycle.

### 2.2. Sampling

This study employed convenience sampling. Eighty-eight prelicensure nursing students, enrolled in the three-unit Chronic Care and End-of-Life Care Theory course at a large public university in San Francisco, CA, USA, were invited to take part in an optional online survey.

### 2.3. Course Design

Table 2 outlines the weekly content of the course under study. As a core requirement in the prelicensure nursing curriculum, this course also fulfills the university’s general education requirement for lifelong learning. The course covers a range of topics, including chronic illness management, palliative and hospice care, communication, cultural competence, legal/ethical issues, and evidence-based interventions [28]. Students enrolled in this course came from both the Bachelor of Science in Nursing (BSN, four-semester) and the Entry-Level Master’s (ELM, six-semester) programs.

### 2.4. Study Design

This study employed an exploratory–descriptive qualitative design, which is suitable for examining specific aspects of participants’ experiences, including their underlying emotions, reflections, and perceptions [29]. Students were asked one open-ended question about how the assignment influenced them both personally and professionally. Data was collected via an anonymous, confidential online survey where participation was voluntary and did not affect course grades. The assignment was introduced in Week 7 and submitted by Week 12. Data collection took place during the Fall 2017 and Spring 2018 semesters.

### 2.5. Data Analysis

Qualitative responses were analyzed using a thematic analysis approach: familiarization with the data, initial coding, categorization, theme development, and reviewing/defining themes [30]. The two authors independently reviewed all open-ended responses and conducted initial open coding to identify meaningful units of text. These units were then grouped into categories through axial coding, and themes were developed inductively through a process of constant comparison. Any discrepancies in coding were discussed until consensus was reached. Given the manageable number of responses, manual coding was conducted using Microsoft Excel. Although qualitative software, such as MAXQDA, was not employed in this study, the use of collaborative manual coding was appropriate to maintain interpretive depth and ensure researcher consensus.

To enhance the trustworthiness of the findings, this study incorporated multiple strategies. Credibility was supported by investigator triangulation, where both authors contributed to data interpretation and theme development. Dependability was maintained by documenting the coding process in an audit trail, including decisions about code refinement. Transferability was promoted by providing detailed descriptions of the course context, assignment structure, and participant demographics.

The analytical process was aligned with Kolb’s Experiential Learning Theory, which guided both the design of the assignment and the interpretation of student reflections. This theoretical framework helped situate student responses within the four stages of experiential learning: concrete experience, reflective observation, abstract conceptualization, and active experimentation.

## 3. Results

Of the 88 students who completed the assignment, 67 (76.1%) participated in the survey. Participants were from two prelicensure programs: the Bachelor of Science in Nursing (BSN; *n* = 35) and the Entry-Level Master’s (ELM; *n* = 32). The sample was predominantly female. The average age was 30.0 ± 7.5 years for BSN students (range, 21–52 years) and 29.5 ± 5.0 years for ELM students (range, 23–48 years).

Table 3 summarizes the themes that emerged from students’ reflections on the AHCD and 5W assignments. Only three students (two ELM and one BSN) reported a prior familiarity with either or both documents before taking the course. A higher proportion of ELM students (63%) reported understanding the documents compared to BSN students (51%). About one-quarter of all participants described the assignment as “interesting” or “emotionally challenging/uncomfortable.” Five students (three BSN and two ELM) shared that the assignment prompted them to confront their own mortality, sparking a reflection on personal wishes and their impact on loved ones.

*“I am interested in completing Five Wishes so that I have complete control over what happens to me in any situation where I can no longer communicate.”*

A subset of students (*n* = 13: 5 BSN, 8 ELM) also reported that the assignment reinforced the importance of nursing advocacy and patient empowerment, prompting them to see their future roles more clearly.

*“Helps me understand what is available for patients to advocate for themselves and what they want for their care.”*

Similarly, 13 students (7 BSN, 6 ELM) noted that the assignments fostered deeper self-reflection, encouraging them to think more seriously about their own wishes and the loved ones in their lives.

*“It made me seriously think about whether I want to be kept alive by a machine. It made me want to make sure my patients have the choice available to them.”*

Some participants reported discomfort in imagining their own end-of-life scenarios or in initiating such “difficult/uncomfortable” conversations with patients:

*“Even though I am happy that I have learned about these two forms, bringing this subject up with a patient is something I may struggle with as a future nurse. I do not know how to approach a patient/client with this form(s). I do not want the patient/client to think I am writing him or her off.”*

One student added the following:

*“I feel that in most hospital settings, these issues have been largely within the scope of the social workers or physicians on the unit.”*

Four students expressed difficulty imagining themselves in end-of-life scenarios, and five indicated that the assignment had little influence on their future professional roles, though they still had positive feelings about the experience.

## 4. Discussion

This exploratory–descriptive qualitative study used Kolb’s experiential learning theory to explore how an AHCD/5W assignment influences nursing students’ understanding of end-of-life care and advance care planning. The findings closely align with earlier studies showing that reflective experience-based learning strengthens students’ confidence in engaging with EOLC documentation and discussion [27,31].

Students described the assignment as a transformative experience that fostered personal insight, professional awareness, and a deeper understanding of patient advocacy. The thematic analysis revealed three key learning outcomes:

1. The recognition of the value of life and mortality.
2. An appreciation of the nurse’s advocacy role in patient-centered EOLC.
3. An enhanced self-awareness of personal values and future care planning.

These outcomes indicate that experiential assignments such as these not only develop clinical knowledge but also build emotional intelligence. The emotional aspect of the experience helps them prepare for challenging difficult clinical conversations with future patients [32].

While several themes emerged during the analysis, it is important to note that a relatively small proportion of students endorsed some response categories. This distribution reflects the open-ended nature of the prompt, which allowed students to focus on personally salient aspects of the assignment. Rather than indicating a lack of impact, the diversity and specificity of the responses suggest a wide range of individual learning outcomes. Even lower-frequency categories, such as discomfort with the topic or challenges imagining one’s own end-of-life, offer meaningful insights into areas where additional educational support may be beneficial. These findings underscore the value of qualitative analysis in capturing nuanced student perspectives that may not be evenly distributed across the sample.

However, three primary challenges emerged:

1. Emotional discomfort and uncertainty about real-life application: Many students reported unease with imagining their own death and dying scenarios or initiating end-of-life conversations with patients, highlighting the importance of ongoing support and clinical practice to build confidence.
2. The need for structured communication training: Students expressed uncertainty regarding when and how to introduce AHCD/5W topics with patients. Nursing education should incorporate role play, scripts, and case study discussions to build these skills and reduce anxiety.
3. Clarifying professional roles in legal and institutional contexts: One student noted confusion about whether ACP responsibilities fall under the nursing scope. This suggests a need for a greater curricular emphasis on legal and ethical frameworks, not to train nurses to complete the ACP documents themselves but to strengthen their ability to engage in meaningful conversations with patients and families, promote understanding, and support informed decision-making [33,34].

It is also worth noting that younger students (those under the age of 25) responded positively, with over 60% demonstrating a heightened awareness of ACP. This supports the value of integrating such assignments early in nursing education, especially for students who may not yet have personal experiences with serious illness or death.

### Implications for Nursing Education and Limitations

In alignment with the American Association of Colleges of Nursing’s *Essentials*, we recommend embedding end-of-life and advance care planning content throughout the undergraduate nursing curriculum [1]. Experiential learning activities such as the AHCD and Five Wishes assignment provide students with an opportunity to engage deeply with complex emotional topics in a reflective and personally meaningful way. These types of assignments can strengthen students’ legal literacy in hospice and palliative care and may support the development of communication skills, ethical reasoning, and cultural sensitivity through guided reflection and discussion.

This study has several limitations that warrant consideration. First, the use of convenience sampling from a single academic institution may limit the generalizability of findings to broader nursing student populations or different educational contexts. While the nursing program is known to be ethnically diverse (comprising 40% White, 30% Asian, and 30% other ethnicities), the demographic data collected did not include specific information on participants’ race or ethnicity, which limits the subgroup analysis.

Second, the participation in the study was voluntary and anonymous, and approximately 24% of students who completed the assignment did not respond to the survey. This introduces the potential for response bias, as those with stronger reactions to the assignment, either positive or negative, may have been more likely to participate.

Third, the survey did not ask students to indicate which document (AHCD or Five Wishes) they completed. As a result, we were unable to conduct comparative analyses based on the assignment type. Similarly, the absence of pre-assignment measures means we cannot determine whether changes in perceptions or understandings were attributable to the assignment itself or pre-existing attitudes.

Despite these limitations, this study offers valuable insights into how experiential learning assignments can encourage personal and professional reflection on end-of-life care among prelicensure nursing students and foster greater awareness of advance care planning.

## 5. Conclusions

The AHCD and Five Wishes assignment was a powerful pedagogical tool that helped prelicensure student nurses engage with the complexities of end-of-life care. Students developed critical insights into their personal values, envisioned themselves in EOL scenarios, and reflected on their responsibility as future nurses. The assignment fostered both professional development and personal growth, making it a meaningful experience within the nursing curriculum.

Beyond its educational value, this assignment has practical implications for nursing education, practice, and policy. By encouraging students to engage personally with end-of-life decisions, the assignment cultivates empathy, ethical reflection, and a readiness to initiate sensitive conversations. These competencies are crucial for real-world clinical settings where nurses often serve as intermediaries in advance care planning.

Findings from this study suggest that experiential ACP assignments can foster student insight and perceived confidence in addressing end-of-life issues. However, the cause-and-effect relationship cannot be confirmed given the study design. Educational programs may consider combining such assignments with role-playing exercises and interdisciplinary simulations to better assess learning outcomes. At the policy level, nursing accreditation bodies may use these insights to promote early, structured exposure to EOLC and ACP as a core competency in prelicensure education.

## Figures and Tables

**Table 1:** The application of Kolb’s Experiential Learning Cycle to the AHCD/5W assignment.

Kolb’s Learning Cycle	AHCD/5W Assignment Application
Concrete Experience	Students complete the AHCD or 5W document as if they were at or near the end of life.
Reflective Observation	Students reflect on the meaning of completing the assignment, both personally and professionally.
Abstract Conceptualization	Students connect their reflections with classroom lectures about EOLC to develop deeper insight.
Active Experimentation	Students apply new knowledge to future practice by assisting patients and families with ACP.

**Table 2:** Weekly content of “chronic care and EOLC theory”.

Week	Content
1	Aging and the state of chronic illness in the United States
2	Theories of aging and age-related physiological changes
3	Dementia, depression, and delirium
4	Geropharmacology, polypharmacy
5	Chronic conditions
6	Chronic care management
7 *	Introduction to hospice and palliative care
8	Pain management in palliative care
9	Symptom management in palliative care
10	Communication in hospice and palliative care
11	Medical aid in dying. Legal and ethical issues in end-of-life care
12 *	Self-care for nurses
13	Final days, final hours: signs/symptoms, nursing care, and interventions
14	Grief, loss, and bereavement
15	Cultural rituals of death and dying (student presentations)
16	Reflection on hospice and palliative care

* The AHCD/5W assignment was introduced in Week 7 and was due in Week 12.

**Table 3:** Student reflections on the AHCD and 5W Assignments.

Categories	BSN (*n*)	ELM (*n*)	Total (*n*/%)
Document learning and understanding	18	20	38 (56.7)
Awareness of document importance	16	11	27 (40.3)
Awareness of patient empowerment	8	5	13 (19.4)
Interesting assignment	10	6	16 (23.9)
Awareness of nurse’s responsibility	17	8	25 (37.3)
Self-reflection	7	6	13 (19.4)
Envisioning own EOL/death	3	2	5 (7.5)
Difficult/uncomfortable assignment/conversation	4	8	12 (17.9)
Opportunity to talk with family about AHCD	4	1	5 (7.5)
Cannot imagine the need for AHCD for myself	2	2	4 (6.0)
No effect, but positive feelings about the assignment	1	4	5 (7.5)

Note: multiple responses allowed. *N* = 67: 35 BSN; 32 ELM. Percentages reflect the frequency within the total sample.

## Data Availability

The data supporting the findings of this study is available upon reasonable request from the first author. The data is not publicly available to protect participant confidentiality.

## Public Involvement Statement

There was no involvement of the public in the design, conduct, reporting, or dissemination of this research.

## Guidelines and Standards Statement

This manuscript was prepared in accordance with the Standards for Reporting Qualitative Research (SRQR) [35], appropriate for exploratory–descriptive qualitative studies.

## Abbreviations

The following abbreviations are used in this manuscript:

5W	The Five Wishes
ACP	Advance Care Planning
AHCD	The Advance Health Care Directive
BSN	Bachelor of Science in Nursing
ELM	Entry-Level Master of Science in Nursing
EOLC	End-of-Life Care
